# Elastin overexpression by cell-based gene therapy preserves matrix and prevents cardiac dilation

**DOI:** 10.1111/j.1582-4934.2012.01560.x

**Published:** 2012-09-26

**Authors:** Shu-Hong Li, Zhuo Sun, Lily Guo, Mihan Han, Michael F G Wood, Nirmalya Ghosh, I Alex Vitkin, Richard D Weisel, Ren-Ke Li

**Affiliations:** a Division of Cardiovascular Surgery and Toronto General Research Institute University Health Network and Department of Surgery Division of Cardiac Surgery, University of TorontoToronto, ON, Canada; b Division of Biophysics and Bioimaging Ontario Cancer Institute/University Health Network and Departments of Medical Biophysics and Radiation Oncology, University of TorontoToronto, ON, Canada

**Keywords:** cell-based gene therapy, elastin, heart failure, bone marrow stromal cells, extracellular matrix

## Abstract

After a myocardial infarction, thinning and expansion of the fibrotic scar contribute to progressive heart failure. The loss of elastin is a major contributor to adverse extracellular matrix remodelling of the infarcted heart, and restoration of the elastic properties of the infarct region can prevent ventricular dysfunction. We implanted cells genetically modified to overexpress elastin to re-establish the elastic properties of the infarcted myocardium and prevent cardiac failure. A full-length human elastin cDNA was cloned, subcloned into an adenoviral vector and then transduced into rat bone marrow stromal cells (BMSCs). *In vitro* studies showed that BMSCs expressed the elastin protein, which was deposited into the extracellular matrix. Transduced BMSCs were injected into the infarcted myocardium of adult rats. Control groups received either BMSCs transduced with the green fluorescent protein gene or medium alone. Elastin deposition in the infarcted myocardium was associated with preservation of myocardial tissue structural integrity (by birefringence of polarized light; *P* < 0.05 *versus* controls). As a result, infarct scar thickness and diastolic compliance were maintained and infarct expansion was prevented (*P* < 0.05 *versus* controls). Over a 9-week period, rats implanted with BMSCs demonstrated better cardiac function than medium controls; however, rats receiving BMSCs overexpressing elastin showed the greatest functional improvement (*P* < 0.01). Overexpression of elastin in the infarcted heart preserved the elastic structure of the extracellular matrix, which, in turn, preserved diastolic function, prevented ventricular dilation and preserved cardiac function. This cell-based gene therapy provides a new approach to cardiac regeneration.

## Introduction

Progressive ventricular remodelling after myocardial infarction (MI) can result in progressive congestive heart failure. After coronary occlusion, the necrosed cardiomyocytes are replaced by fibroblasts and the infarcted myocardium becomes fibrotic scar tissue containing predominantly type III collagen. Because the intraventricular pressure consistently stretches the stiff, non-elastic, infarcted tissue with each contraction, the scar expands and the left ventricular (LV) chamber dilates with time, initiating the spiral of increased ventricular chamber stiffness and decreased cardiac function [1, 2].

The elastic properties of tissues are essential to maintain their structural and biological function. Elastin is a major fibrous protein in the extracellular matrix of organs and tissues that exhibit stretch and recoil, such as vessel walls and articular ligaments [3]. When the tissue is stretched, the elastin molecule is elongated, and when the stretching force is released, the molecule returns to its more stable random-coil structure and maintains the organ structure. Most cells, such as fibroblasts, endothelial cells and smooth muscle cells, synthesize and secrete glycoproteins to form a microfibrillar scaffold, on top of which tropoelastins, the soluble precursors of elastin, are assembled and covalently cross-linked by lysyl oxidase into the resilient elastin polymer [4–8].

Restoration of the elastic properties of the infarct region can prevent progressive cardiac dilation and deterioration of ventricular function following an MI [9, 10]. Myogenic cell transplantation has been suggested as a means of modifying the infarct region from a stiff, fibrotic tissue to an elastic, contractile tissue [11–13]. Our previous studies have shown that somatic or stem cells injected into the infarcted myocardium survive after implantation and form tissue [11, 14]. Unfortunately, the low retention and limited survival of the implanted cells limits this approach, and most studies have demonstrated that paracrine signalling effects of the engrafted cells are responsible for the improvement in cardiac function. We also demonstrated that genetic modification of implanted cells to overexpress tissue inhibitors of matrix metalloproteinases (TIMPs) limited matrix disruption and improved cardiac function post-MI [15]. Therefore, we hypothesized that increasing elastin gene expression in the infarct region could preserve distensibility and compliance to prevent scar expansion, ventricular dilation and cardiac dysfunction.

In the present study, we transduced primary cultured rat bone marrow stromal cells (BMSCs) with a high-efficiency adenoviral vector to overexpress the full-length elastin gene and found that implantation of autologous BMSCs overexpressing elastin prevented post-MI ventricular remodelling and improved cardiac function. This study provides a new approach to restore the promise of cardiac regeneration by cell transplantation.

## Materials and methods

### Cloning of elastin cDNA

A full-length human lung elastin cDNA was isolated, compared with sequences in GenBank (GenBank ID: NM_001081755.1; Fig. S1), and cloned into an adenovirus (Ad) vector under control of the cytomegalovirus (CMV) promoter (Fig. S2), as described in the Supplementary Methods. Expression of human elastin in the adenoviral system was confirmed by Western blotting with polyclonal rabbit anti-tropoelastin (Elastin Products Company, Inc., Owenswille, MO, USA).

### Cell transduction

Primary rat BMSC cultures were generated as previously described [12, 16]. Cells from passages 2–5 were used for the study. Cells were transduced (MOI 50) with the Ad-CMV-elastin or Ad-CMV-green fluorescent protein (GFP) construct or with empty vector (control) for 2 hrs. The viral supernatant was removed, and serum-free fresh medium was added for 2–4 days.

### Immunochemical staining and RT-PCR

BMSCs were seeded and grown on slides and then transduced with the Ad-CMV-elastin or Ad-CMV-GFP construct. At 48 hrs post-transduction, the cells were fixed, washed, blocked and probed with polyclonal rabbit anti-human elastin at a dilution of 1:50, followed by goat anti-rabbit-Alexa 568 (Invitrogen, Burlington, ON, Canada) at a dilution of 1:200. The cells were then stained with FITC-conjugated phalloidin (Invitrogen) at a dilution of 1:50. The nuclei were counterstained with DAPI (Sigma-Aldrich, Oakville, ON, Canada), and the cells were visualized by fluorescence microscopy.

RT-PCR was performed using the SuperScript III system (Invitrogen) to evaluate human and rat elastin gene expression *in vitro* and *in vivo*. Primer pairs were specific for human elastin (sense: 5′-gcc att cct ggt gga gtt cct gga-3′; antisense: 5′-acc gca cct gca gac act cct aag-3′) or rat elastin (sense: 5′-ctt cct ggt gga gtt ccc ggt gga-3′; antisense: 5′-ccg atg cca cca ata cca ccg aca-3′). Reverse transcription was carried out at 50°C for 30 min. For PCR, we used 30 cycles at 95°C for 30 sec., 55°C for 1 min. and 72°C for 1 min. The RT-PCR products were visualized on 1% agarose gels using ethidium bromide. GAPDH was amplified as a reference.

### Myocardial infarction and cell transplantation

All animal procedures were approved by the Animal Care Committee of the University Health Network, and all animals received humane care in compliance with the *Guide for the Care and Use of Laboratory Animals* (National Institutes of Health, 1996).

Adult female Lewis rats were anaesthetized using isoflurane (2%) during mechanical ventilation. The left coronary artery ligation technique was used to induce an MI and generate an infarct scar in the heart [1, 2, 9, 13]. Animals in the sham group had open-chest operation, but no artery ligation. Seven days after ligation, cardiac function was evaluated by two-dimensional echocardiography. Only rats exhibiting fractional shortening between 20% and 40% were included in this study. The selected rats were randomly divided into three groups: Iscove's modified Dulbecco's medium (medium control), BMSCs transduced with Ad-CMV-GFP (BMSC-G, cell control) and BMSCs transduced with Ad-CMV-elastin (BMSC-elastin). Medium alone or medium mixed with BMSCs (3 × 10^6^ cells/rat) was directly injected into the central region (one injection) and border region (one injection in each of two different sites) of the infarct using a 28G needle 7 days after MI. We consider 1 week after ligation the optimal time for cell transplantation as the acute inflammatory response has subsided, but the scar is not yet mature with dense collagen deposition.

### Elastin protein measurement

For determination of *in vitro* elastin expression in BMSCs transduced with Ad-CMV-elastin, an antibody specific for human tropoelastin was used. Tropoelastin protein was detected by Western blot and quantitated by densitometry, with GAPDH as a loading control.

### Myocardial matrix assessment

Left ventricular sections from all groups were collected 9 weeks after cell transplantation. To assess the elastic structure of the tissue, sections were stained using the Verhoeff Van Gieson method and visualized with a Nikon Ti-Eclipse microscope. Three random fields of view were acquired and averaged for the positive pixel area of elastin fibres in the remote, border and infarct regions of each animal using ImageJ software. Elastin fibre–coated blood vessels were identified and digitally removed. Elastin fibre–positive pixel area was measured and expressed as the percentage of total possible pixels.

### LV morphological assessment

The LV volume was measured using image analysis of pressure-fixed heart specimens, as previously described [17]. Briefly, after completing the functional measurements, hearts were quickly excised, fixed at an intraventricular pressure of 30 mmHg and then cut into 2-mm thick slices. Heart slices were photographed, and computed planimetry was used to calculate morphometric parameters for each heart. Scar area was calculated as a percentage of the LV wall (LVW) surface area, as follows: (epicardial scar length)/(epicardial LVW length) × 100. Scar thickness was presented as an average of five measurements of LVW thickness equally distributed within the scar region.

### Tissue birefringence measurements

Tissue birefringence was measured *ex vivo* as previously described [18]. Briefly, the hearts were excised, fixed and sectioned into 1-mm slices. Birefringence was assessed through measurements of linear retardance produced by anisotropic tissue structures exposed to polarized light. Polarized light was transmitted through the sections, and the output state of the light's polarization was measured for varying input polarization states. From these measurements, the polarizing effects of the tissue, including birefringence, were calculated. The measurable effect of birefringence is termed ‘retardance’ and was used in this study as a measure of tissue organization.

### Analysis of cardiac function

Echocardiographic examinations were performed on treated rats, using an ACUSON Sequoia C256 System (Siemens, Mississauga, ON, Canada) with a 15L8 transducer (13 MHz), prior to ligation, prior to cell transplantation and every 2 weeks for 10 weeks after ligation. LV end-diastolic and end-systolic diameters and areas were measured, and per cent fractional shortening was calculated as previously described [19].

End-point functional assessments were performed using Millar pressure and conductance catheters (Millar Instruments, Houston, TX, USA), as previously described [19]. Real-time pressure–volume (P–V) loops were constructed and adjusted for parallel conductance [19]. Load-sensitive haemodynamic parameters (ejection fraction, end-systolic volume, end-diastolic volume) and LV P–V relationships were analysed with the PVAN software package (Millar Instruments). Load-independent indices of systolic function (maximal systolic elastance) and diastolic function (end-diastolic pressure-volume relationship [EDPVR], or end-diastolic elastance) were calculated from P–V loops constructed during vena cava occlusion.

### Statistics

Data are presented as mean ± S.E.M. Time course analyses were carried out using two-way ANOVA, comparisons among groups were carried out with one-way ANOVA, and Tukey's multiple comparison test was used to detect data points at which differences between groups reached statistical significance. The P–V relations were compared by analysis of covariance.

## Results

### Cells transduced with elastin cDNA expressed and secreted elastin *in vitro*

**Fig 1:**
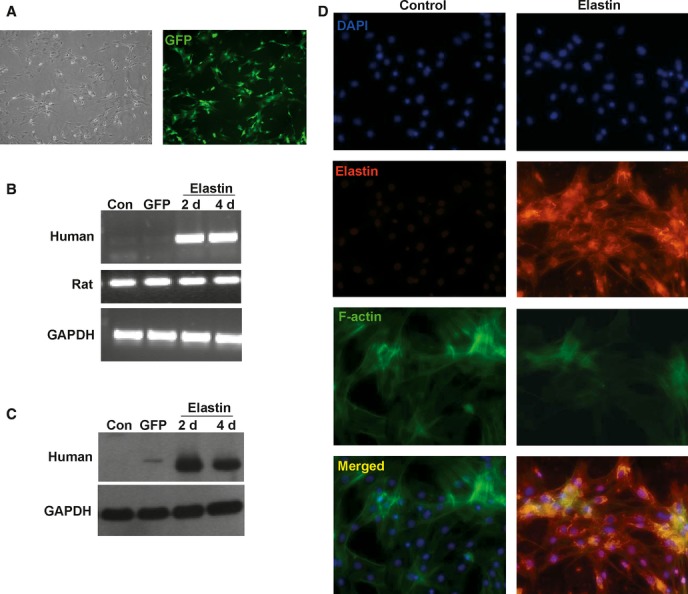
Recombinant elastin expression in rat BMSCs. (A) Cultured rat BMSCs were effectively transduced with the adenovector, as indicated by GFP fluorescence in Ad-CMV-GFP transduced cells at day 4 (40× magnification). (B) Human elastin mRNA in the BMSCs was evaluated by RT-PCR 2 and 4 days after transduction, with primer sets specific for human and rat elastin. (C) Western blotting with an antibody specific for human tropoelastin confirmed the expression of elastin protein in the Ad-CMV-elastin transduced rat BMSCs. (D) Immunostaining with DAPI, anti-human elastin antibody and FITC-conjugated phalloidin (for F-actin) revealed no detectable human elastin in the culture of BMSCs transduced with empty vector (left panels). However, BMSCs transduced with Ad-CMV-elastin (right panels) showed a network of human elastin molecules (200× magnification). As the strong elastin staining in the Ad-CMV-elastin group overshadowed the F-actin staining, we enhanced the contrast/brightness of the F-actin staining to show that most of the elastin-staining cells were F-actin positive. (Con = empty vector; GFP = Ad-CMV-GFP; representative of three experiments).

Rat BMSCs were first transduced with Ad-CMV-GFP to test the transduction efficiency. The cells expressed GFP at detectable levels 2 days after transduction, and the efficiency was approximately 90–95% (Fig. 1A). To confirm expression of the human elastin gene and evaluate its effect on endogenous rat elastin expression, RT-PCR was performed. Rat BMSCs transduced with Ad-CMV-elastin expressed human elastin mRNA 2 and 4 days after transduction, but no human elastin mRNA was found in control or Ad-CMV-GFP cells (Fig. 1B). Induction of the human elastin gene in rat BMSCs did not alter the expression of endogenous elastin as no changes in rat elastin gene expression were observed among the control, Ad-CMV-GFP or Ad-CMV-elastin cells (Fig. 1B). In agreement with the mRNA expression profile, BMSCs transduced with Ad-CMV-elastin expressed the elastin protein, but no human elastin was found in the control or Ad-CMV-GFP cells (Fig. 1C). Immunochemical staining with an antibody against human elastin showed that elastin was successfully deposited into the extracellular matrix of rat cells 2 days after Ad-CMV-elastin transduction and formed an extended network of elastin fibres, which was absent in the control cells transduced with empty vector (Fig. 1D).

### Transplanted elastin-expressing cells formed an elastin network and preserved the myocardial architecture *in vivo*

**Fig 2:**
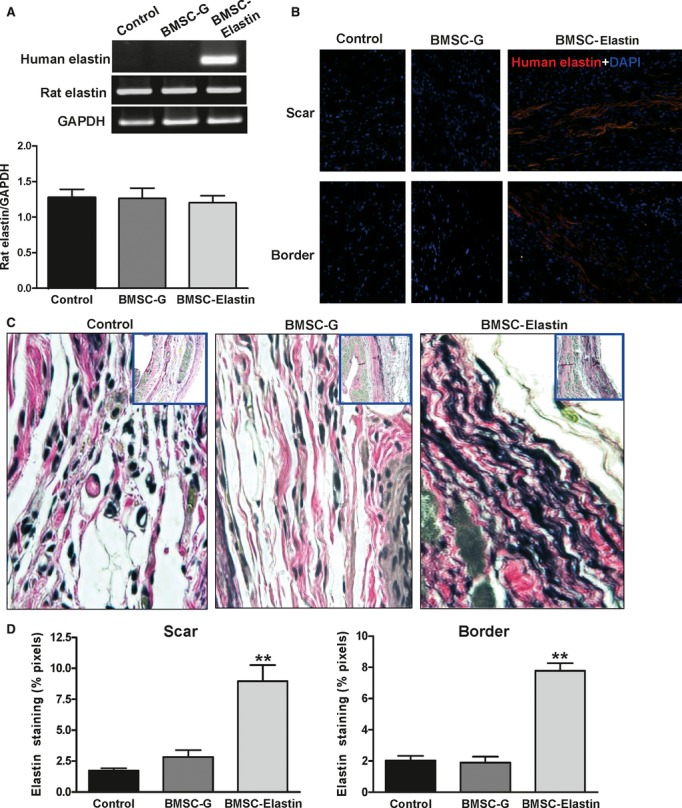
Expression of elastin in the infarcted myocardium. (A) Seven days after ligation, BMSCs transduced with Ad-CMV-elastin (BMSC-elastin) or Ad-CMV-GFP (BMSC-G) were injected into the central region and border zone of the infarct. Medium alone served as control. Human and rat elastin mRNA in the infarct and border regions was evaluated by RT-PCR 7 days after injection, with primer sets specific for human and rat elastin. GAPDH served as a loading control. The BMSC-elastin group expressed human elastin mRNA, but no human elastin mRNA was found in the medium control or BMSC-G groups. Induction of the human elastin gene in transduced rat BMSCs did not alter the expression of endogenous elastin as no significant changes in rat elastin gene expression were observed among the medium control (*n* = 3), BMSC-G (*n* = 3) or BMSC-elastin (*n* = 5) groups. (B) Seven days after ligation, BMSCs transduced with Ad-CMV-elastin (BMSC-elastin) were injected into the infarct. Medium alone served as control. Human elastin in the infarct and border regions was evaluated by immunofluorescent staining 7 days after injection. Representative micrographs (200× magnification) show that the BMSC-elastin group expressed human elastin protein, but no human elastin was found in the medium control and BMSC-G groups. Nuclei were stained with DAPI. (C) Verhoeff Van Gieson staining of mid-papillary transverse myocardial sections (400× and 40× magnification) of infarcted myocardium shows elastin fibres in the medium control, BMSC-G and BMSC-elastin groups 9 weeks after cell transplantation. (D) The pixel area positive for elastin was significantly greater in the scar and border zone of the BMSC-elastin group compared with medium control and BMSC-G groups (*n* = 5 per group for the scar, *n* = 6 per group for the border zone) (**P < 0.01 *versus* control).

One week after coronary artery ligation, we implanted genetically modified rat BMSCs expressing either human elastin (BMSC-elastin) or GFP (BMSC-G) into the infarcted rat myocardium. Medium without cells served as the control. RT-PCR was used to evaluate human and rat elastin mRNA expression in the infarct and border regions 7 days after injection. The BMSC-elastin group expressed human elastin mRNA, but no human elastin mRNA was detected in the medium control or BMSC-G groups. Expression of the endogenous rat elastin was not significantly altered by the induction of human elastin (Fig. 2A). To further confirm human elastin deposition in the infarcted myocardium, frozen sections of the hearts were immunohistochemically stained using an antibody against human elastin. As illustrated in Fig. 2B, human elastin molecules were identified in the myocardium of the BMSC-elastin group, but not in the control groups.

At 9 weeks after implantation, Verhoeff Van Gieson staining for elastic tissues demonstrated that the infarct scar and border region of the control and BMSC-G groups contained few elastin fibres (Fig. 2C). In contrast, there was a greater accumulation of elastin in these regions of the BMSC-elastin group (Fig. 2D). The elastin fibres were configured in a wavy pattern, resembling that of normal elastin in an arterial wall, and formed an extensive elastin network around the infarcted area.

**Fig 3:**
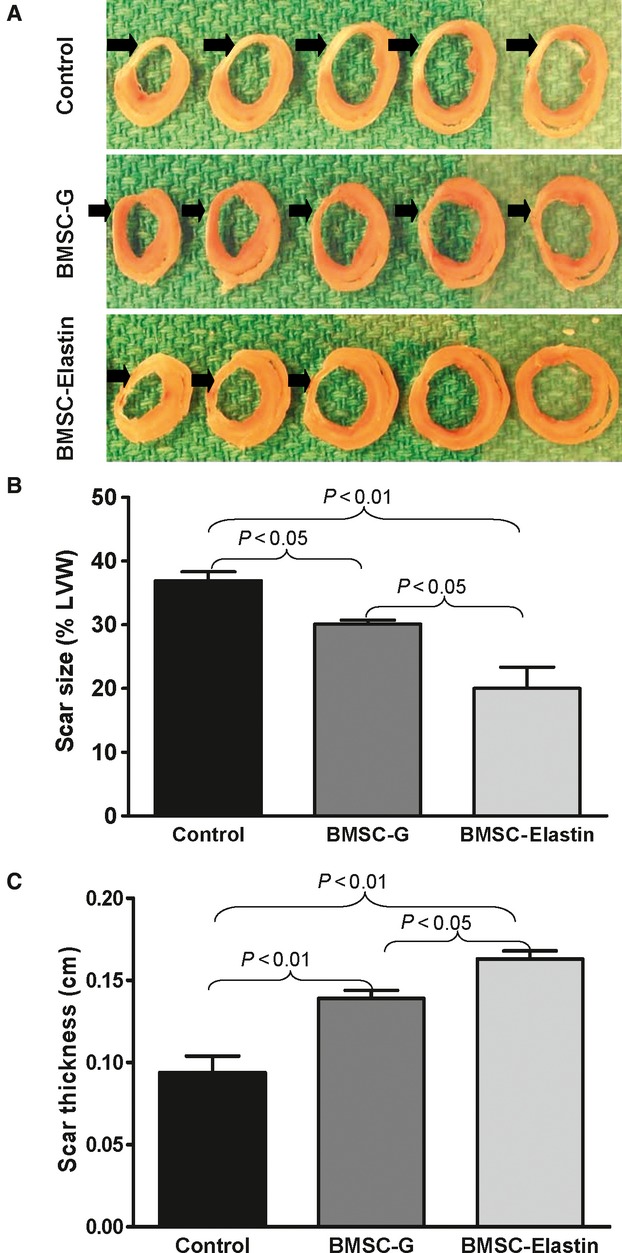
Infarct scar characterization. (A) Sections of infarcted rat hearts show fibrotic scar tissue in the LV free wall (arrows) 9 weeks after cell transplantation. (B, C) Scar size (*n* = 5 per group) and thickness (*n* = 4 per group) were evaluated by computed planimetry. The BMSC-elastin group had the smallest and thickest scars compared with the other two groups.

Morphological examination of the hearts 9 weeks after cell implantation (Fig. 3A) revealed larger (*P* < 0.05) and thinner (*P* < 0.01) scars in the medium control group compared with the BMSC-G group (Fig. 3B and C). The BMSC-elastin group had the smallest and thickest scars relative to the medium control (*P* < 0.01, both indices) and BMSC-G (*P* < 0.05, both indices) groups, suggesting that elastin overexpression *in vivo* stabilized the infarct region.

**Fig 4:**
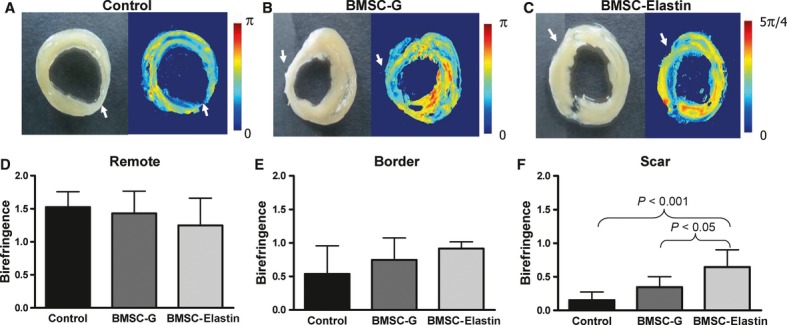
Tissue organization of the infarcted myocardium. (A–C) Sections of infarcted rat hearts (left panels) were used for birefringence imaging analysis (right panels) 9 weeks after cell transplantation. White arrows indicate scar area. Myocardial birefringence (retardance) was determined by polarized light transmission through tissue sections and provides an index of tissue structural organization. (D–F) Myocardial birefringence (measured in radians) of the infarcted myocardium showed no significant group differences in the remote region (D, *n* = 3 per group) or border zone (E, *n* = 3 per group); however, within the infarct scar (F, *n* = 4 per group), birefringence in the BMSC-elastin group was significantly higher than that in the medium control and BMSC-G groups, indicating maintenance of tissue organization with elastin overexpression.

The variation in the organization of the extracellular matrix after cell implantation was evaluated by quantification of myocardial birefringence (Fig. 4), through measurements of linear retardance produced by anisotropic tissue structures exposed to polarized light. The polarized light anisotropy measurements are primarily indicative of tissue organization and are not sensitive to tissue composition. Although there was no difference among groups in the remote region or border zone, myocardial birefringence in the scar region was significantly greater in the BMSC-elastin group than in the other two groups (*P* < 0.001 *versus* medium control; *P* < 0.05 *versus* BMSC-G). This increase in birefringence (retardance) indicates an increase in tissue organization and suggests that elastin overexpression preserved the tissue structural integrity of the infarcted myocardium.

### Expression of elastin prevented cardiac dysfunction

**Fig 5:**
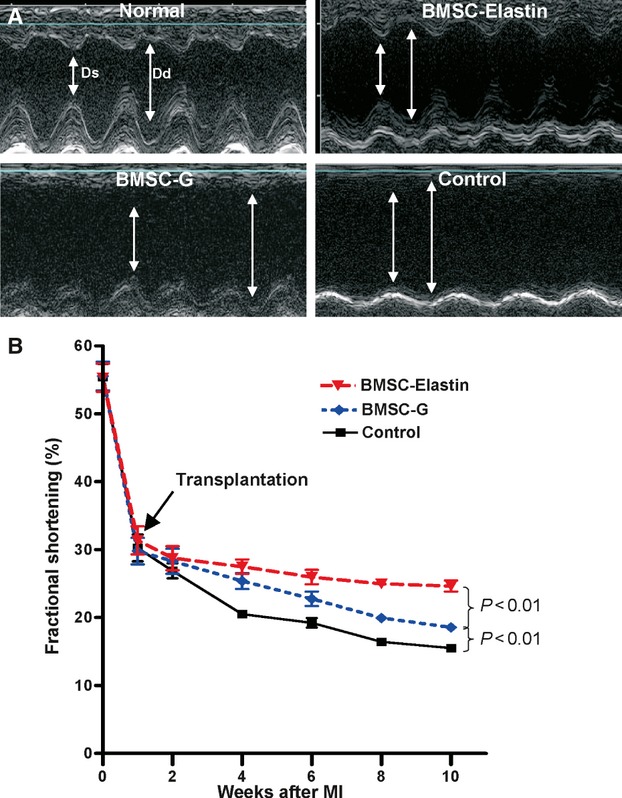
Echocardiographic analysis of cardiac function. (A) Echocardiographic M-mode traces of rat hearts before MI (normal) and 10 weeks after MI. Ds = end-systolic dimension, Dd = end-diastolic dimension. (B) Prior to and 1 week after MI, there was no difference in fractional shortening among groups (*n* = 8 per group). However, 10 weeks post-MI, the BMSC-G group showed significant preservation of fractional shortening compared with the control group, whereas the BMSC-elastin group showed significantly greater fractional shortening compared with either the medium control or BMSC-G group.

Cardiac function was evaluated with echocardiography (Fig. 5A) prior to ligation, prior to cell transplantation and every 2 weeks for 10 weeks following MI. Cardiac function decreased dramatically in all three groups 1 week after MI (Fig. 5B). However, 9 weeks after cell transplantation (10 weeks post-MI), the BMSC-G group demonstrated significant preservation of fractional shortening compared to the medium control group (*P* < 0.01). A significantly greater preservation of fractional shortening was observed in the BMSC-elastin group compared to the BMSC-G group (*P* < 0.01).

**Fig 6:**
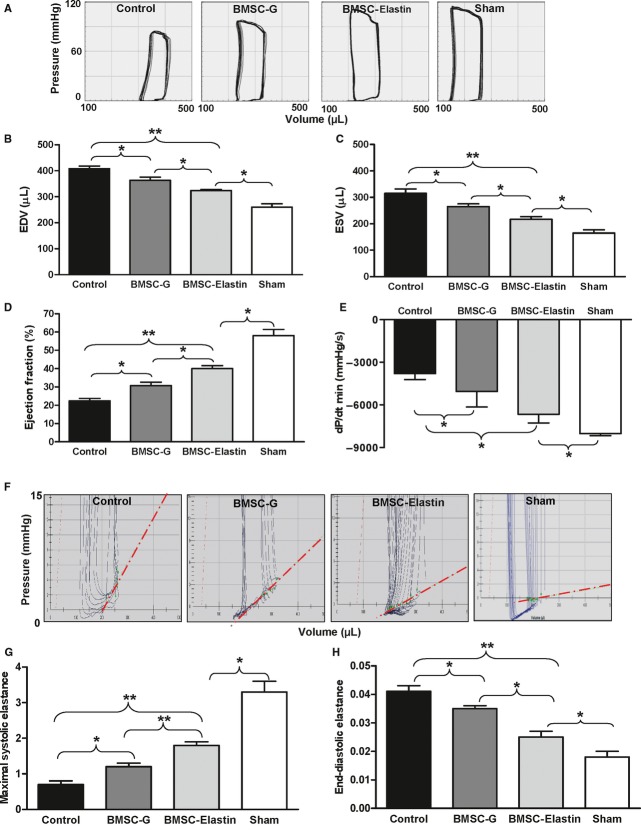
Pressure–volume analysis of cardiac function. (A) Representative P–V loops of rat hearts 9 weeks after cell transplantation. A sham non-ligated control depicts normal cardiac function. The BMSC-G and BMSC-elastin groups had smaller ventricular volumes than the medium control group. (B, C) The BMSC-elastin group had significantly smaller end-diastolic volume (EDV) and end-systolic volume (ESV) than the BMSC-G group (*n* = 4 per group). (D, E) The load-dependent index of systolic function (ejection fraction) was significantly greater in the BMSC-elastin group than the BMSC-G group, which was better than the medium control group. Diastolic function (d*P*/d*t*_min_) was significantly better in the BMSC-elastin group *versus* the other two groups (*n* = 5 per group). (F) Representative P–V loops during vena cava occlusion demonstrate diastolic function and the position and slope of the end-diastolic pressure–volume relationship (EDPVR, red dotted line). (G, H) The load-independent indices of both systolic and diastolic function (maximal systolic elastance and end-diastolic elastance) were significantly improved in the BMSC-elastin group compared with the BMSC-G group, which was better than the medium control group (*n* = 4 per group). Elastin overexpression resulted in a more compliant left ventricle. (*P < 0.05, **P < 0.01).

Cardiac function and ventricular volumes were also evaluated using a P–V catheter 9 weeks after cell transplantation (Fig. 6A). A sham non-ligated control group was used for comparison and indicated that none of the indices of cardiac function for the three treatment groups were restored to normal levels. The medium control group had the largest end-diastolic and end-systolic volumes. Cell implantation (BMSC-G) resulted in smaller end-diastolic volume (*P* < 0.05) and end-systolic volume (*P* < 0.05) compared to medium alone (Fig. 6B and C). Overexpression of elastin further reduced systolic and diastolic volumes (*P* < 0.05 *versus* BMSC-G, both indices).

With respect to load-sensitive indices of cardiac function, the BMSC-elastin group had greater ejection fraction than the BMSC-G group (*P* < 0.05; Fig. 6D), which was better than the medium control group (*P* < 0.05), and d*P*/d*t*_min_ was better preserved in the BMSC-elastin group compared to the other two groups (*P* < 0.05; Fig. 6E). The load-independent assessment of diastolic compliance demonstrated greater diastolic stiffness (diastolic P–V loops were shifted up and to the left) in the medium control group, with better preservation of distensibility in the BMSC-G group and greatest diastolic elastance in the BMSC-elastin group (diastolic P–V curves were shifted down and to the right with a lower slope; *P* < 0.05; Fig. 6F). The load-independent indices of systolic function (maximum systolic elastance; Fig. 6G) and diastolic function (end-diastolic elastance; Fig. 6H) were better preserved in the BMSC-G group than the medium control group (*P* < 0.05, both indices), and best preserved in the BMSC-elastin group (*P* < 0.01 *versus* medium control, both indices).

## Discussion

Stem cell therapy of infarcted hearts promised to restore ventricular function by replacing lost cardiomyocytes with functioning contractile elements. However, cell implantation has been shown to improve ventricular function only by paracrine signalling [20]. Cell-based gene therapy permits augmentation of this signalling by providing the appropriate cytokines in the optimal amounts and at the optimal times to improve angiogenesis, modify matrix remodelling and increase stem cell homing. However, the survival of both implanted and recruited myogenic cells in the infarct region (and border zone) has been limited [21]. We have previously demonstrated the potential of attenuating progressive cardiac dilation and ventricular dysfunction by restoring the elastic properties of the infarct region through overexpression of an elastin gene fragment [9, 10]; however, the efficacy achieved with the elastin fragment was limited. Therefore, in the current study, we investigated whether the addition of elastic components by overexpression of the full-length elastin gene in addition to cell therapy could modulate matrix changes after an MI and induce a greater recovery of ventricular function than we previously observed.

Variations in the human elastin gene from different tissues indicate that there are tissue-specific isoforms of tropoelastin, which may possess different biological properties [22]. We cloned a full-length elastin cDNA from a human lung cDNA library, which was confirmed by extensive direct nucleotide sequencing as being identical to elastin transcript variant 5 (Figs. S1 and S2). Compared with the elastin gene found in the human aorta, our specific lung isoform lacked exon 23 and did not possess the rs2071307 polymorphism [23] found in both aorta and skin [22, 24].

The human lung elastin gene was recombined into an adenoviral vector and successfully expressed in primary cultured rat BMSCs. Matrix modulation with increased elastin deposition in the extracellular matrix was observed in these cell cultures. We injected these genetically modified BMSCs into infarcted rat hearts in the belief that improvements in cardiac function due to cell transplantation could be further enhanced by introducing the full-length elastin gene into the transplanted cells, hence increasing the elasticity of the scar tissue after an MI. We observed elastin mRNA and protein in the rat hearts injected with BMSCs expressing human elastin. In both our *in vitro* and *in vivo* studies, we used RT-PCR to evaluate rat endogenous elastin expression and believe that the expression of human elastin did not significantly alter the endogenous elastin gene expression. However, a limitation of our study is that we do not have real-time PCR data to draw a definitive conclusion. We confirmed the presence of more elastic fibres in the infarct scar of the BMSC-elastin group by Verhoeff Van Gieson staining. In addition, these elastin fibres were configured in a wavy pattern, forming an extensive elastin network around the infarcted area. In the medium control and BMSC-G groups, the infarct scars had substantially fewer organized elastin fibres. We speculate that expression of elastin preserved the organized extracellular matrix and prevented adverse matrix modulation. Future studies would be of interest to determine the effects of elastin overexpression on other extracellular molecules, such as collagen.

Morphometric analysis of the infarct scars revealed that scar expansion and thinning were reduced in the BMSC-elastin group, supporting the observation that matrix disruption was reduced by the expression of elastin in the transplanted cells. Subsequent investigation confirmed the preservation of structure as myocardial birefringence in the scar region of the BMSC-elastin hearts was significantly higher than that of the other two groups, indicating a better aligned and organized extracellular matrix. Linear birefringence results from spatial anisotropy of optical refractive indices in tissue and can be used as a metric of tissue organizational structure [25], with significant potential for monitoring regenerative therapies in the heart [26]. This new polarized light–based imaging modality provides direct evidence of tissue characteristics within the myocardial infarct region [27]. Together with our observation of more elastic fibres in the infarct scar of the BMSC-elastin group, this technique provides convincing evidence of altered elasticity in the infarcted myocardium and therefore preservation of distensibility with the overexpression of elastin by the engrafted cells.

Matrix modulation plays an important role in maintaining ventricular geometry and function. We have previously shown that the loss of matrix structure and spatial architecture in *Timp3*^−/−^ mice was associated with decreased ventricular distensibility, chamber dilation, and systolic and diastolic dysfunction [28] and that reduced TIMP3 protein in cardiomyopathic hamsters was associated with progressive adverse remodelling of the myocardial matrix and heart failure [29]. These changes were similar to the results found in hearts from patients with end-stage cardiomyopathy [29]. Enhancing cell therapy with the *Timp3* gene reversed these effects and improved the recovery of ventricular function [15].

Decreased diastolic distensibility and compliance are important contributors to heart failure. Nearly half of the patients presenting with symptoms of congestive heart failure exhibit a near normal LV systolic function at rest [30, 31], and their symptoms are believed to be the result of diastolic dysfunction. Morphological examination demonstrated excessive myocardial fibrosis and disruption of the elastin components of the matrix architecture [32]. Diastolic dysfunction is therefore an important contributor to progressive heart failure, and altering diastolic compliance may prevent decompensation of systolic ventricular performance.

The passive compliance of the ventricular wall is as important to ventricular filling as active myocardial relaxation. Other determinants of diastolic function in addition to myocardial stiffness include wall thickness, chamber geometry and the timing of relaxation. The d*P*/d*t*_min_, the maximal velocity of ventricular pressure fall during diastole, is a load-dependent index of diastolic compliance. In our study, the BMSC-elastin group had the smallest d*P*/d*t*_min_ value. The EDPVR provides direct load-independent quantitative evidence of chamber stiffness. These data demonstrated that elastin overexpression in transplanted BMSCs significantly prevented the increase in diastolic stiffness seen in the other groups. Preservation of matrix structure and diastolic function may have contributed to the improved systolic function and cardiac performance associated with the transplantation of elastin-transduced BMSCs. Compared with our previous study using the elastin gene fragment [10], overexpression of the full-length elastin gene was associated with better cardiac function. Both the morphological observations and the functional measurements suggest that the additional elasticity of the infarct region may increase ventricular recoil in response to the stresses of contraction and reduce the stresses on the ischaemic heart to prevent scar thinning and dilation. Therefore, preservation of diastolic compliance may have prevented adverse matrix remodelling of the injured heart.

In the current study, we demonstrated that matrix modulation by gene-enhanced cell transplantation preserved regional elasticity through overexpression of the full-length elastin gene. Elastin overexpression stabilized tissue structure, reduced scar expansion and improved cardiac function, especially LV diastolic function. These observations suggest that increasing the elasticity of the infarct scar will produce significant physiological benefits. The improved ventricular function following implantation of BMSCs transduced with the elastin gene suggests that elastin overexpression may be an important method to improve the results of cell transplantation.

## Supporting information

**Fig. S1**. Characterization of the elastin gene. (**A**) The human lung elastin cDNA is 2115 base pairs and contains 33 exons encoding 705 amino acids. (**B**) The elastin genes cloned from lung, aortic smooth muscle cells [1] and skin fibroblasts [2] have 90% homology. Nucleotide A at position 1263 reported in human aortic and skin fibroblast cDNA clones is replaced by G in the lung elastin cDNA clone, resulting in the substitution of glycine for serine. The lung elastin gene also lacks exon 23. (**C**) PCR using pairs of specific primers designed to amplify the missing exon confirmed its absence. Although we identified other exons using primer pairs 3F/5R and 2F/5R, the cDNA containing exon 23 could not be identified. (MW = molecular weight; representative of three experiments).

**Fig. S2.** Recombination of elastin cDNA into an adenovector. (**A**) The full-length elastin cDNA was recombined into a replication-defective Ad vector (pAdEasy-1, 30 kb). (**B**) The right lane is the digested products of the shuttle vector, which contained the elastin insert. This confirms the successful ligation of the gene into the shuttle vector. The positive clone (left lane) represents successful recombination of the elastin insert into the adenovector. (**C**, **D**) Transduction of AD-293 cells with Ad-CMV-GFP had 100% efficiency. (**E**) Non-transduced AD-293 cells. (**F**) After transduction of Ad-CMV-elastin into AD-293 cells, the elastin molecules in the cell lysate increased. (**G**) Immunoblot analysis suggests the expression of the human tropoelastin gene in Ad-CMV-elastin transduced cells. (Representative of three experiments).
