# Supplementary figures and images for: Elastin overexpression by cell-based gene therapy preserves matrix and prevents cardiac dilation
Source: J Cell Mol Med. 2012 Sep 26;16(10):2429–39. doi: 10.1111/j.1582-4934.2012.01560.x (PMC3823437; doi:10.1111/j.1582-4934.2012.01560.x)

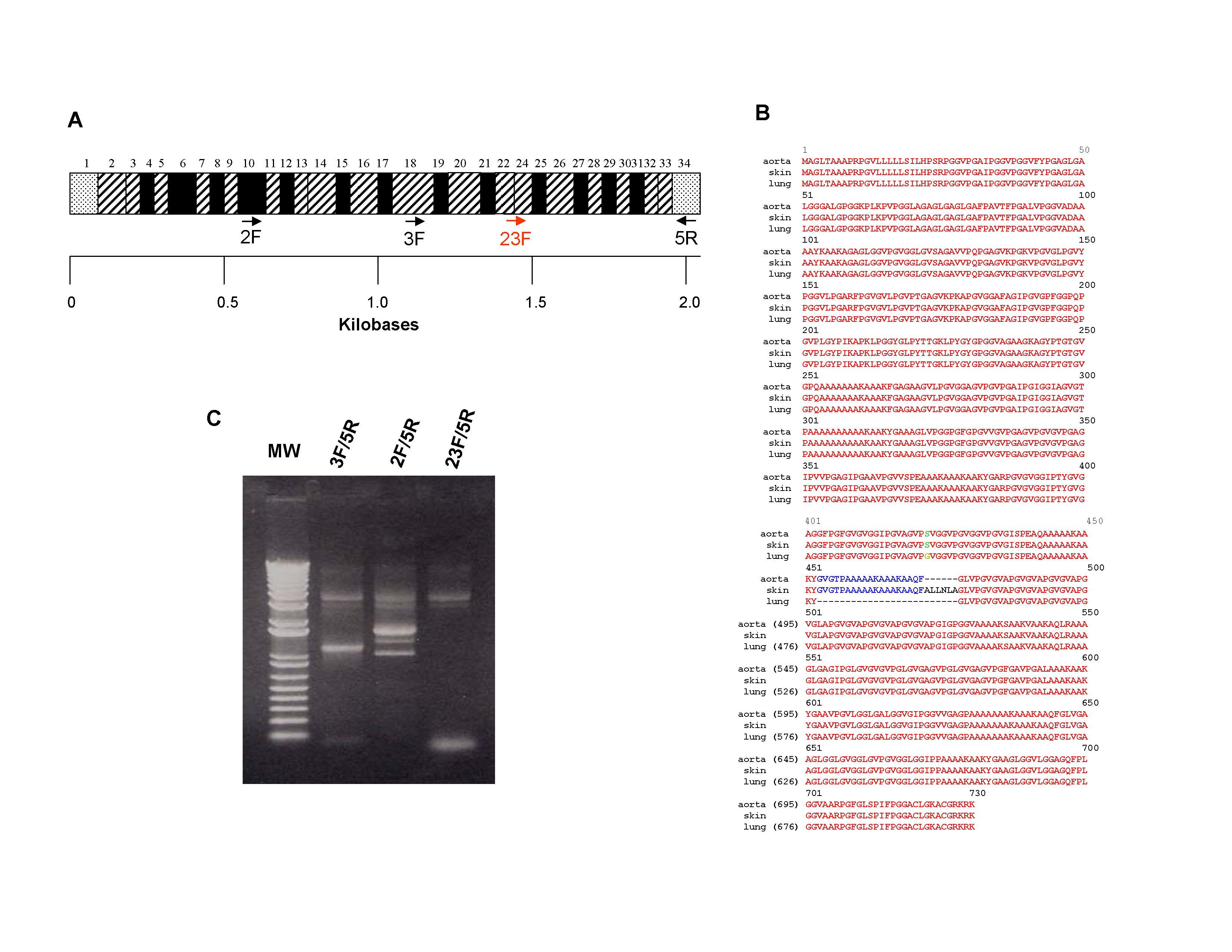

Supplement: Supplementary file 1 [file jcmm0016-2429-SD1.tif]

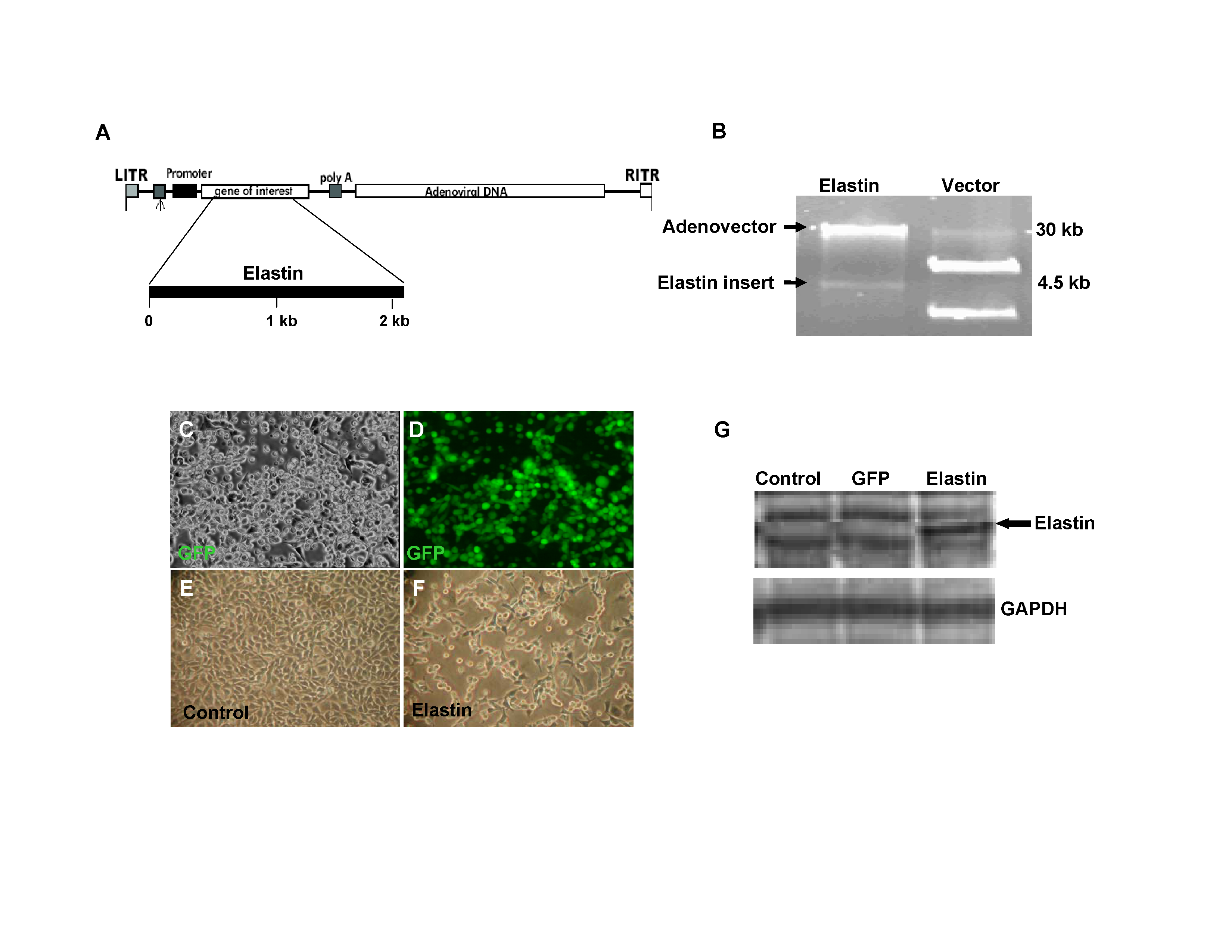

Supplement: Supplementary file 2 [file jcmm0016-2429-SD2.tif]
